# Bothropic snakebite in preterm pregnancy: Antivenom and clinical outcome in mother and newborn in Cúcuta, Colombia

**DOI:** 10.7705/biomedica.7595

**Published:** 2025-08-11

**Authors:** Diana Marcela Pava, Marisol Galindo, Juan Felipe Bedoya, Guadalupe Osorio, Mario Javier Olivera, Santiago Ayerbe, José Leonardo Gómez, Helver Guiovanni Rubiano

**Affiliations:** 1 Dirección de Producción, Instituto Nacional de Salud, Bogotá, D. C., Colombia Dirección de Producción Instituto Nacional de Salud Bogotá, D. C. Colombia; 2 Hospital Universitario Erasmo Meoz, Cúcuta, Norte de Santander, Colombia Hospital Universitario Erasmo Meoz Cúcuta Norte de Santander Colombia; 3 Dirección de Investigación en Salud Pública, Instituto Nacional de Salud, Bogotá, D. C., Colombia Dirección de Investigación en Salud Pública Instituto Nacional de Salud Bogotá, D. C. Colombia; 4 Facultad de Ciencias de la Salud, Universidad del Cauca, Popayán, Colombia Universidad del Cauca Facultad de Ciencias de la Salud Universidad del Cauca Popayán Colombia; 5 Dirección General, Instituto Nacional de Salud, Bogotá, D. C., Colombia Dirección General Instituto Nacional de Salud Bogotá, D. C. Colombia

**Keywords:** Snake bites, pregnancy, Colombia, mordeduras de serpientes, embarazo, Colombia

## Abstract

Snakebite envenomation remains a neglected public health problem in many tropical and subtropical countries. It mainly affects rural populations and has a higher incidence in men. Most cases have been reported in Africa, Latin America, and Asia. More than 300 species of snakes have been identified in Colombia, of which around 18% are of medical importance. This fact places the country as the third with the highest number of cases in the region, with 6,231 reported by 2023.

Snakebite envenomation in pregnant women is a rare event, and it implies a higher risk of fetal and neonatal death. We report the case of a newborn with neonatal hypoxia and fetal distress, resulting from a cesarean section of a 22-year-old primigravida at 36 weeks of gestation after an ophidian accident involving a bite in the dorsum of her left hand. The newborn was admitted to the intensive care unit in critical condition and with progressive clinical deterioration. However, following the timely administration of antivenom and mechanical ventilation, the infant showed a remarkable recovery and was discharged after only 12 days of hospitalization.

We underline the need to improve the availability of antivenoms and to strengthen pharmacovigilance systems to ensure their effectiveness and safety. In conclusion, this clinical case highlights the importance of an early consultation, the availability and prompt administration of the antivenom, and the expertise of healthcare workers in managing this event in pregnant women and neonates.

## Resumen

Las mordeduras de serpiente son un problema de salud pública desatendido en muchos países tropicales y subtropicales. Afectan principalmente a las poblaciones rurales y se presentan con mayor frecuencia en hombres. La mayoría de los casos se registran en África, Latinoamérica y Asia. En Colombia, se han identificado más de 300 especies de serpientes, de las cuales aproximadamente el 18 % son de importancia médica. Este panorama ubica al país como el tercero con mayor número de casos en la región, con 6.231 reportes durante el 2023.

El ofidismo en mujeres gestantes es un evento infrecuente, y se asocia con un mayor riesgo de muerte fetal y neonatal. Se presenta el caso de un neonato con hipoxia y sufrimiento fetal, producto de una cesárea en una mujer de 22 años, primigestante, con feto único de 36 semanas, tras una mordedura de serpiente en el dorso de la mano izquierda.

El recién nacido ingresó a la unidad de cuidados intensivos en estado crítico y con signos de deterioro progresivo. No obstante, la administración oportuna del antiveneno y el soporte vital mediante asistencia respiratoria mecánica, se observó una recuperación notable y fue dado de alta después de tan solo 12 días de hospitalización.

Este caso resalta la necesidad de optimizar la disponibilidad de antivenenos y fortalecer los sistemas de farmacovigilancia para garantizar su efectividad y seguridad. En conclusión, se destaca la importancia de una atención temprana, y de la disponibilidad y el uso rápido del antiveneno, así como la experiencia y el conocimiento del personal sanitario sobre el manejo de este tipo de eventos en mujeres embarazadas y neonatos.

Snakebites are one of the main neglected public health problems in several tropical and subtropical countries. Globally, an estimated 5.4 million people are bitten by snakes each year, with an alarming number of envenomation cases ranging from 1.8 to 2.7 million ^1^. These bites may have devastating consequences, causing between 81,410 and 137,880 deaths and three times as many amputations and other permanent disabilities. Most of these incidents occur in Asia, Africa, and Latin America. In Asia, up to 2 million individuals experience snakebite envenomation annually, while in Africa, from 435,000 to 580,000 people need treatment for snakebites per year. In the Americas, there are approximately 57,000 cases of snakebite per year, with a fatality rate of 0.6%. These cases affect women, children, and rural workers in poor communities from low- and middle-income countries, particularly in those with poor health systems and limited medical resources ^1^.

Colombia, due to its favorable eco-epidemiological conditions for the presence of snakes, faces a significant public health challenge related to snakebites. It is considered the third Latin American country with the highest incidence, after Brazil and Mexico. According to available national records, more than 300 species of snakes have been identified across Colombia, inhabiting altitudes ranging from sea level to 3,500 meters above sea level. Of these species, approximately 18% are venomous ^2^. Since 2021, the incidence of snakebites has shown a steady increase. By 2023, 6,149 cases and 33 deaths were reported to the Colombian public health surveillance system (*Sistema Nacional de Vigilancia en Salud Pública*, SIVIGILA), including 105 cases in pregnant women with an average gestational age of 24 weeks ^3^.

In particular, the *Bothrops* genus is one of the most medically important in central and northern South America, responsible for 50% to 80% of snakebites in the region ^2^. In the southeast of Colombia, three species -*B. asper, B. rhombeatus,* and *B. ayerbei-* are responsible for the most severe snakebite cases. These species are phylogenetically related and show remarkable differences in the pathophysiological profile of their envenomations ^4^.

Snakebite envenomations in pregnant women can have serious maternal and fetal effects, including miscarriage, placental abruption, premature delivery, fetal malformations, and maternal, fetal, or neonatal death. Limited literature on this topic evidence the need for a better understanding of the consequences of this event in the mother/child binomial ^5^.

In Colombia, very specific cases have been described in case reports. SIVIGILA includes variables indicating whether a woman was pregnant at the time of the ophidian accident. This information is available in technical reports and anonymized databases. Any member of the community can have access to them.

In cases of envenomation, the timely administration of antivenom is crucial to reduce morbidity and mortality, restore hemostatic function, and reduce tissue damage ^6^. However, few documented cases report the administration of antiophidian serum in newborns, highlighting a significant gap in the current knowledge and management of this issue. This report describes a case of snakebite envenomation in a pregnant woman, detailing the situation management and the outcomes for the mother and child.

## Case presentation

**Figure 1:**
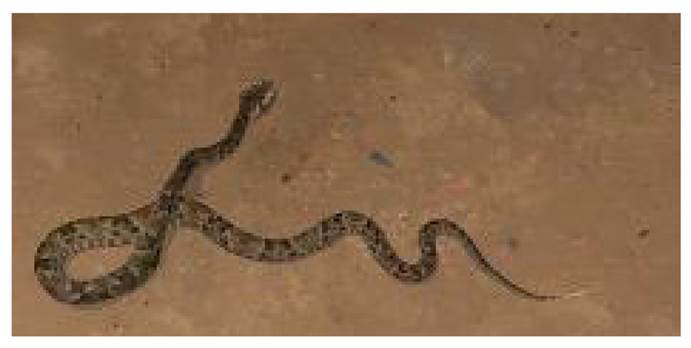
A juvenile specimen of Bothrops atrox, a medically important viperid species in South America

**Figure 2:**
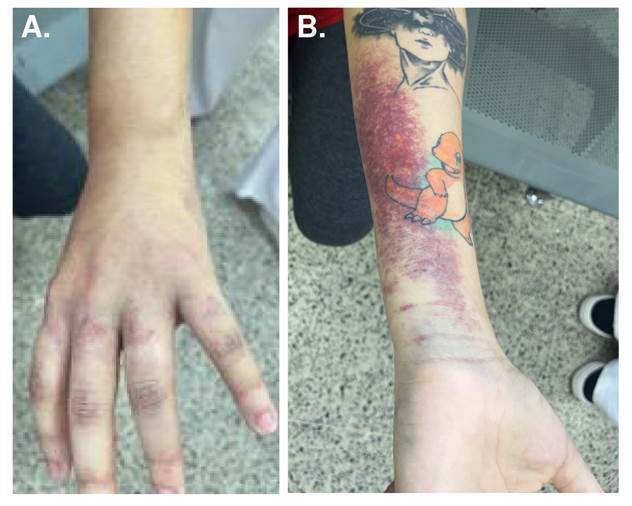
Clinical presentation of snakebite envenomation in a pregnant woman. A) Dorsal view of the left hand, where the snakebite occurred. B) Extensive ecchymosis and subcutaneous hemorrhage on the left forearm

We report the case of a 22-year-old woman with 36 gestational weeks who was admitted to the emergency department following a snakebite on the dorsum of her left hand by a species of medical importance identified as a juvenile specimen of *B. atrox* (figure 1). The patient presented clinical symptoms of 35 minutes of evolution characterized by edema and intense pain (figure 2A and 2B), compatible with snake envenomation. Initial management included the administration of two vials of polyvalent antiophidic serum from the *Instituto Nacional de Salud de Colombia* and 200 mg of hydrocortisone. No signs of respiratory distress, tachycardia, or diaphoresis were observed in the mother. However, fetal monitoring revealed persistent tachycardia of up to 200 beats per minute, suggestive of fetal distress. Given this situation, an emergency cesarean section was performed. Additional management included toxicology assessment and laboratory tests to evaluate the condition of the mother and child.

**Figure 3:**
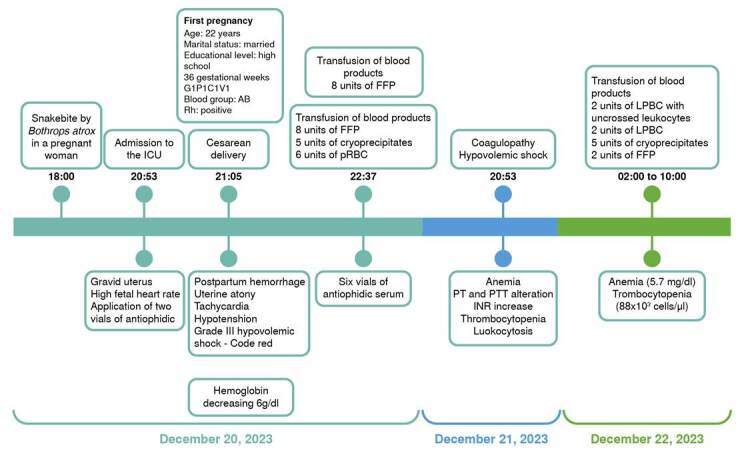
Chronological progression of clinical manifestations in a pregnant woman following a snakebite

FFP: Fresh frozen plasma; pRBC: Packed red blood cells; LPBC: Leukocyte-poor blood components; PT: Prothrombin time; PPT: Partial thromboplastin time; INR: International normalized ratio

After surgery, the patient was in fair general condition, with a heart rate of 107 and a hypotonic uterus in the immediate postoperative period. She developed grade III hypovolemic shock and was transferred to the intensive care unit for pain control, transfusion of blood products, and administration of six additional vials of antiophidian serum. Blood products included eight units of fresh frozen plasma, six units of packed red blood cells, and five units of cryoprecipitates. Afterwards, laboratory tests showed fibrinogen consumption and prolonged coagulation times. Chest X-ray and hepatobiliary ultrasound showed bilateral basal infiltrates and a right-sided pleural effusion. The patient was discharged after four days of clinical management (figure 3).

The newborn was delivered by emergency cesarean section due to fetal tachycardia. The fetus was in cephalic presentation, with the umbilical cord loose around the neck. At birth, the infant had hypotonia, sporadic gasping, cyanosis, and an Apgar score of 3 at minute 1, and 7 at 5 minutes. Noninvasive positive pressure ventilation was initiated, resulting in heart rate normalization and improved coloration -oxygenation- within the first minute. However, the baby did not show spontaneous breathing. Within four minutes, he experienced the bradycardia again but recovered quickly. At 7 minutes, he began to breathe spontaneously but with persistent wheezing and maintained an adequate heart rate and oxygenation.

The physical examination was standard, with a heart rate of 156 beats per minute. The neurological evaluation revealed initial depression, followed by improved muscle tone and reflexes. The newborn weighed 2,580 g, measured 49 cm, and had a head circumference of 29.5 cm, a thoracic circumference of 29 cm, and an abdominal circumference of 26 cm. The infant was referred to the intensive care unit due to the risk of respiratory failure and side effects associated with the maternal history of the ophidian accident. As signs of respiratory distress progressed, the newborn required non-invasive ventilation. Diagnostic assessments included chest X-ray, laboratory tests, transfontanelle brain ultrasound, total abdomen ultrasound, and toxicology evaluation.

Laboratory results revealed prolonged coagulation times, leading to the administration of polyvalent antiophidian serum produced by the Colombian *Instituto Nacional de Salud*. Six ampoules were diluted in 40 ml of 0.9% saline solution. The serum was administered for 90 minutes. The infusion was initiated at a very slow rate of one drop per minute during the first 20 minutes. In case of no adverse reactions, the remaining volume was administered over the next 70 minutes. Results of laboratory tests are shown in (table 1).

**Table 1:** Temporality of laboratories of the neonate, child of a pregnant woman with snakebite.

Tests		12/21/23 4:18	12/21/23 12:28	12/22/23 18:49	12/22/23 20:01	12/23/23 11:43	12/24/23 7:02	12/25/23 12:40	12/25/23 10:15	12/26/23 5:45	12/26/23 17:01	12/27/23 13:11	12/28/23 1:55	12/28/23 5:00	12/29/23. 16:53	12/30/23 6:00	12/31/23 11:04	01/01/24 6:00	01/02/24 6:01
C-reactive protein (mg/L)		0.10	-	-	-	23.00	-	10.73	-	-	-	-	-	-	-	-	7.14		Discharge
Hemogram																			
Leukocytes (10^3^ /μl)		13.22	-	-	-	13.34	-	11.13	-	-	-	-	-	-	-	-	11.47	-	-
Hemoglobin (g/dl)		15.6	-	-	-	10.4	-	15.1	-	-	-	-	-	-	-	-	14.1	-	-
Hematocrit (%)		43.6	-	-	-	29.8	-	43.7	-	-	-	-	-	-	-	-	40.3	-	-
Platelet count (10 ^3^ /μl)		415	-	-	-	179	-	133	-	-	-	-	-	-	-	-	561	-	-
Coagulation panel																			
	PT sec	17.1	-	21.4	14.1	15.3	-	12	-	-	-	-	-	-	-	-	-	-	-
	INR	1.5	-	1.88	-	1.34	-	1.06	-	-	-	-	-	-	-	-	-	-	-
	PTT	81.2	-	50.1	36.4	43.4	-	32.9	-	-	-	-	-	-	-	-	-	-	-
	Fibrinogen (mg/dl)	-	164	-	-	322	-	-	-	-	-	-	-	-	-	-	-	-	-
Blood chemistry																			
Glucose (mg/dl)		155	-	94	-	77	107	91	-	94	88	62	-	70	81	90	70	70	-
BUN (mg/dl)		-	16.45	-	-	-	-	-	-	-	-	-	-	-	-	-	-	-	-
Sanguineous urea (mg/dl)		-	35.2	-	-	-	-	-	-	-	-	-	-	-	-	-	-	-	-
Creatinine (mg/dl)		-	0.88	-	-	-	-	-	-	-	-	-	-	-	-	-	-	-	-
Procalcitonin		-	-	-	-	-	-	-	-	-	-	-	-	-	-	-	0.13	-	-
Total bilirubin		-	-	-	-	-	-	-	-	16.95	-	-	4.73	-	-	-	-	-	-
Direct bilirubin		-	-	-	-	-	-	-	-	1.34	-	-	0.78	-	-	-	-	-	-
Indirect bilirubin		-	-	-	-	-	-	-	-	15.61	-	-	3.95	-	-	-	-	-	-
Sodium (mEq/L)		-	133	139	-	142	-	-	-	-	-	-	-	-	-	-	-	-	-
Potassium (mEq/L)		-	5.7	4.1	-	3.7	-	-	-	-	-	-	-	-	-	-	-	-	-
Chlorine (mEq/L)		-	106	110	-	111	-	-	-	-	-	-	-	-	-	-	-	-	-
Calcium (mg/dl)		-	1.3	0.86	-	1.17	-	-	-	-	-	-	-	-	-	-	-	-	-
Microbiology																			
	CSF-Gram	-	-	-	-	-	-	-	-	-	Negative	-	-	-	-	-	-	-	
	CSF-Culture	-	-	-	-	-	-	-	-	-	Negative	-	-	-	Negative	-	-	-	
Blood cultures		Negative	-	-	-	-	-	-	Negative	-	-	-	-	-	-	Negative	-	-	
Urinalysis		-	-	Normal limits	-	-	-	-	-	-	-	-	-	-	-	-	-	-	
Transfusion of blood components																			
pRBC (poor in leukocytes, ml)	-	-	-	-	-	48	-	-	-	-	-	-	-	-	-	-	-	-	-

PT: Prothrombin time; INR: International normalized ratio; PTT: Partial thromboplastin time; BUN: Blood urea nitrogen; CSF: cerebrospinal fluid; pRBC: packed red blood cells - No laboratory tests were done.

On the third day of hospitalization, the infant developed acute anemia, requiring a red blood cell transfusion. As coagulation times stabilized, no additional doses of antivenom were necessary. On the fourth day, the baby was extubated and initiated nasal non-invasive ventilation. On the sixth day, due to the patient’s moderate icteric skin (Kramer score = 2-3), the medical team indicated intermittent phototherapy and bilirubin measurement. On the eighth day, invasive ventilation was suspended. On days 9-11, laboratory controls were performed. Finally, on day 12, the baby was discharged.

Resolution 8430 of 1993, issued by the Ministry of Health, establishes scientific, technical, and administrative standards for health research in Colombia. According to Article 11 of this resolution, which classifies research by risk level, this study is considered risk-free. This work only applied documentary research techniques and methods; it did not involve any intervention or intentional modification of the biological, physiological, psychological, or social variables of the participants. The information collected was stored anonymously and protected through the informed consent of the participants.

Throughout the study process, the four internationally accepted basic principles of research were considered: beneficence, justice, nonmaleficence, and autonomy.

## Discussion

Snakebite in pregnant women is a rare event. Between 2019 and 2022, 82 cases of ophidian accidents in pregnant women were reported to SIVIGILA, corresponding to 0.39% of the cases notified for this period. The average age was 24 years (range: 13 to 46), and the average gestational age was 24 weeks (range: 2 to 41). Of the reported pregnant women, 53.7% were from Antioquia, Córdoba, Chocó, Bolívar, La Guajira, and Arauca. No deaths were recorded in this population ^7^.

The severity, lethality, and maternal and fetal complications depend on the degree of envenomation and the gestational age of the fetus ^8^. The decision to use an antivenom relies on the clinical classification and degree of envenomation, the clinical condition of the patient, the involved snake species, and the possible toxic effects of the venom. Most antivenoms are administered intravenously under close monitoring, allowing for early detection and treatment of related adverse reactions ^9^.

Snake bites can cause systemic effects, such as nausea, vomiting, increased heart rate, abdominal pain, bleeding, and necrosis. Treatment includes antivenom administration and management of complications that may arise. Careful patient monitoring and observation is essential to ensure proper care.

According to the study by Nascimento *et al.*, 70 to 90% of the predominant snake species in some areas of Brazil belong to the *Bothrops* genus (commonly known as lanceheads), accounting for about 25,000 snakebite cases ^7^. In the present case report, the pregnant woman reported having been bitten by a specimen of this genus, specifically *B. atrox*.

Reviews conducted in different Latin American countries have also reported cases of snakebites in pregnant women: up to 213 cases with a lethality of 4% and fetal loss of 20% ^10^. In Brazil, 274 cases were reported in pregnant women, with a frequency of severe envenomation of 7.9% and a case fatality rate of 0.4%. The frequency of perinatal death was 5.6%, including 2.8% fetal deaths and 2.8% neonatal deaths. In Mexico, only a few cases have been documented in pregnant women, with four cases registered in the first semester of 2020.

Overall, this case was exceptional compared to typical snakebite envenomation, as it involved vertical transmission, resulting in fetal tachycardia and a subsequent hemotoxic condition after birth. It is noteworthy how quickly the pregnant woman sought medical attention, which is essential in these situations. In addition, this report emphasizes the availability and timely administration of specific antivenom by trained healthcare workers. The expertise of the medical staff was crucial to identify the envenomation in the mother and neonate and quickly administer the antivenom.

Snake venom is a complex mixture of proteins and non-protein substances. In *Bothrops* species, “the main effects reported are related to coagulation disorders, hemorrhage and tissue damage” ^11^. Most venoms are primarily composed of metalloproteases, which are involved in anticoagulant processes, hemorrhages and nephrotoxicity. Locally, envenomation may also cause edema, pain, and blister formation ^11^.

In this case report, some of the previous clinical manifestations were observed in the pregnant woman, such as edema, ecchymosis, grade III hypovolemic shock, and alterations in coagulation times; the fetus exhibited tachycardia and prolonged coagulation times. The fetal symptoms may be explained because the placenta forms a functional interface between maternal and fetal circulation. It facilitates gas and metabolite exchange, secretes hormones, and acts as a barrier between the two immune systems, allowing fetal survival in the uterus ^12^.

Hatakeyama *et al.* analyzed venoms of *Bothrops* species *in vivo* and *in vitro* in each life stage and identified differences in their clinical manifestations, severity, and lethality. They found that young snakes produce greater alterations in coagulation times than adult snakes. This finding is consistent with the present case, in which a juvenile specimen was identified as the cause of envenomation ^13^.

Literature about antivenom administration in neonates is scarce. Saavedra-Orozco *et al.* presented the clinical case of a pregnant woman with a twin pregnancy of 36 weeks who experienced an ophidian accident by *Bothrops* spp. One of the neonates had prolonged coagulation times without alterations in the platelet count or active bleeding. Therefore, the authors decided to give her a dose of antiophidian serum, which improved coagulation times. After five days of hospitalization, the infants were discharged ^14^. The clinical management of the mother and babies was similar to that of the current report: Here, the newborn received six ampoules of antivenom due to her clinical condition and risk of bleeding.

## Conclusions

Published literature on the administration of antivenom in newborns remains limited, likely due to the rarity of snakebites in pregnant women -an event associated with high morbidity and mortality for the fetus and mother. Health institutions should ensure availability and timely access to specific antivenoms and must have health workers trained in snakebite cases to establish an early diagnosis, implement appropriate measures to treat the envenomation, and safeguard the life of the mother-child binomial, as well as education in rural communities about snakebite in pregnant women.

In cases of envenomation in pregnant women, the first recommendation is to administer antivenom as soon as possible. The same applies if the newborn’s clinical manifestations are suggestive of envenomation. In the case presented, we highlight the clinical effectiveness of the antivenom to correct the hemotoxic condition in the mother and the newborn. This study provides a solid basis for future research on ophidian accidents in pregnant women and neonates, addressing the safety of antivenom in newborns and the long-term impact on the child’s neurological development. These results are relevant as they contribute to snakebite understanding and management.

## Recommendations

Coagulation time is required in primary health care as part of the paraclinical tests to evaluate hemotoxicity, categorize the severity of snakebite envenomation, and serve as an indicator for clinical followup. Currently, medical staff performs the dry tube hemotoxicity test (also known as “the all-or-none test”). However, its sensitivity and specificity are debated as envenomation is a dynamic event, which implies that clinical manifestations and coagulation disturbances may vary over time.

It is important that protocols and management guidelines for ophidian accidents include specific recommendations for populations with distinct pharmacokinetic and pharmacodynamic characteristics, such as pregnant women and newborns. Variations in distribution volumes, plasma proteins, free drug fraction, and the multicompartmental model of the mother-child binomial influence decision-making in an emergency context.

Ophidian accidents in pregnant women are considered serious events that requires the articulation of emergency medical systems and referral of the mother-fetus binomial to the highest level of care available. Strict clinical follow-up is essential, including monitoring fibrinogen degradation products to assess the progression of hemotoxicity and guide therapeutic decisions.
